# Identification and Characterization of miRNAs in Self-Rooted and Grafted *Malus* Reveals Critical Networks Associated with Flowering

**DOI:** 10.3390/ijms19082384

**Published:** 2018-08-13

**Authors:** Na An, Sheng Fan, Yang Yang, Xilong Chen, Feng Dong, Yibin Wang, Libo Xing, Caiping Zhao, Mingyu Han

**Affiliations:** 1 College of Horticulture, Northwest A&F University, Yangling 712100, Shannxi, China; anna_luck@163.com (N.A.); likessd@126.com (S.F.); chen_xilong@outlook.com (X.C.); dong-feng@nwafu.edu.cn (F.D.); kobewyb@163.com (Y.W.); libo_xing@nwafu.edu.cn (L.X.); cpzhao403@163.com (C.Z.); 2 College of Life Science, Northwest A&F University, Yangling 712100, Shaanxi, China; 3 Innovation Experimental College, Northwest A&F University, Yangling 712100, Shaanxi, China; yang543052239@nwsuaf.edu.cn

**Keywords:** *Malus × domestica*, grafting, microRNA, flowering, transcription

## Abstract

Grafting can improve the agricultural traits of crop plants, especially fruit trees. However, the regulatory networks and differentially expressed microRNAs (miRNAs) related to grafting in apple remain unclear. Herein, we conducted high-throughput sequencing and identified differentially expressed miRNAs among self-rooted Fuji, self-rooted M9, and grafted Fuji/M9. We analyzed the flowering rate, leaf morphology, and nutrient and carbohydrate contents in the three materials. The flowering rate, element and carbohydrate contents, and expression levels of flowering genes were higher in Fuji/M9 than in Fuji. We detected 206 known miRNAs and 976 novel miRNAs in the three materials, and identified those that were up- or downregulated in response to grafting. miR156 was most abundant in Fuji, followed by Fuji/M9, and then self-rooted M9, while miR172 was most abundant in M9, followed by Fuji/M9, and then Fuji. These expression patterns suggest that that these miRNAs were related to grafting. A Gene Ontology (GO) analysis showed that the differentially expressed miRNAs controlled genes involved in various biological processes, including cellular biosynthesis and metabolism. The expression of differentially expressed miRNAs and flowering-related genes was verified by qRT-PCR. Altogether, this comprehensive analysis of miRNAs related to grafting provides valuable information for breeding and grafting of apple and other fruit trees.

## 1. Introduction

Grafting, in which a scion is joined to a rootstock, is an important method to improve agricultural traits. The scions and rootstocks synergistically regulate plant growth and regulation. Grafting is a useful method to fix or improve agronomic traits in different plants [1]. On the one hand, scions can be weakly or strongly affected by rootstocks. For example, different rootstocks can affect plant height, timing of fruit maturity, and/or stress defenses [2]. For example, the tree height of Nagafu No. 2 was higher when grafted onto T337 rootstocks than onto M111 rootstocks [3]. Watermelon grafted onto two different rootstocks (Japanese pumpkin and black seed pumpkin) showed different degrees of cold resistance [4]. On the other hand, rootstocks can also be affected by different scions. For example, Hongguan and Hongyu apple varieties grafted onto the same M13 rootstock showed differences in their nitrogen (N) and magnesium (Mg) contents [3]. Such studies have shown that scions and rootstocks interact with each other to improve agronomic traits, and these potential interactions can differ among plant species and cultivars.

The development of molecular biology and sequencing technologies has allowed researchers to explore the potential mechanisms of the interaction between scions and rootstocks. Putative messengers between scions and rootstocks have been identified by sequencing, especially important microRNAs (miRNAs) [5]. miRNAs are a class of 18- to 24-nt noncoding RNAs that have been identified as major regulators of eukaryotic gene expression in recent years. miRNAs were first discovered in 1993, in *Caenorhabditis elegans* [5]. By 2001, miRNAs had been confirmed to be widely present in the animal kingdom [6,7], and a large number of miRNA families have also been found in plants [8,9]. One miRNA can have several to hundreds of target genes [10,11]. Many studies have shown that miRNAs play an important role in regulating various growth and development stages, such as flower induction, hormone signal conduction, defense against stress, and morphology of roots, stems, and leaves [12,13]. miRNAs also play an important role in the interaction between scions and rootstocks. Previous studies have shown that miR395, miR398, and miR399 in the phloem are highly associated with stress, and that miR395 and miR399 are able to move from scions to rootstocks and affect the expression levels of their own encoding genes [14]. The transporter of miR399 was found to regulate the expression of *PHO2*. Other studies have shown that miR172 can move from source to sink organs in *Nicotiana* [15] and that miR156 functions as a phloem-mobile signal to affect agricultural traits in potato [16].

The most important miRNAs in the regulation of flowering are those in the miR156 and miR172 families [17]. In *Arabidopsis*, miR156 functions together with its target, SQUAMOSA promoter-binding protein-like (*SPL*), to regulate flower induction, and there are similar relationships between mi159 and *MYB*, and miR172 and *AP2* [18,19,20]. Various miRNAs have been found to be involved in shoot bending–mediated flower induction and in vegetative growth in apple, and these miRNAs together with hormones and/or flowering-related genes control flowering [20]. Functional analyses have shown that ectopic expression of Md-miRNA156h or Md-miR172e resulted in later or earlier flowering, respectively, in *Arabidopsis* [21,22].

Apple is an important economic tree that is grown worldwide. Nagafu No. 2 (Fuji) is one of the most widely cultivated varieties, and accounts for 72% of the apple cultivation area in China. Apple flower induction is an important issue that has attracted much attention from researchers. Hormone- or sugar-mediated flower induction has been well studied in apple [12]. M9, a widely used dwarfing rootstock, was better in disease resistance and other agronomical traits and was widely used in apple planting in China. Self-rooted Fuji has a long vegetative stage, a low flowering rate, and poor tree growth, but Fuji grafted onto M9 rootstock does not have these problems. These substantial differences in phenotype as a result of grafting have not been well characterized in apple. Because miRNAs are known to move between scions and rootstock and play an important role in flower induction, we used high-throughput miRNA sequencing to explore the potential molecular regulation mechanisms of scions and rootstocks. Comprehensive morphologic, physiologic, and molecular analyses were conducted for self-rooted Fuji, self-rooted M9, and grafted Fuji/M9 (hereafter referred to as Fuji, M9, and Fuji/M9) to compare their different physiologic responses and potential regulatory networks. The results of this study provide information about the miRNA-mediated response to grafting that will be useful for further research on apple and other fruit trees.

## 2. Results

### 2.1. Differences in Phenotypes among Three Apple Materials

**Figure 1:**
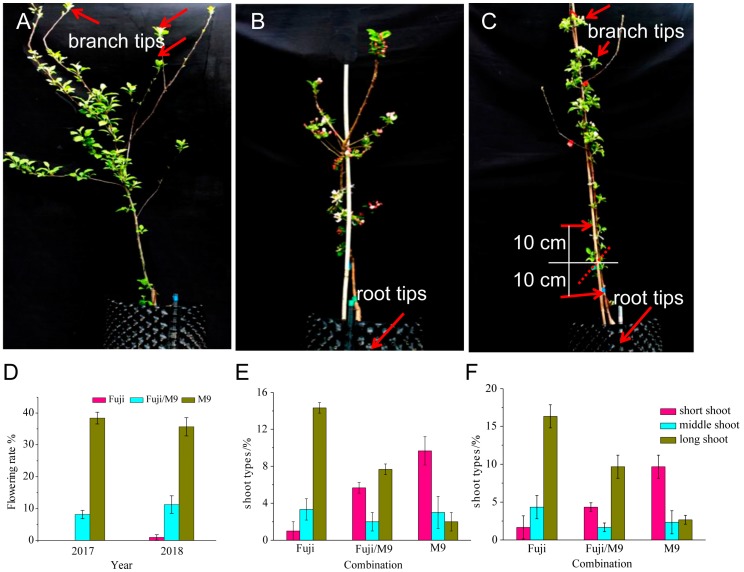
Phenotypic and physiologic differences of self-rooted Fuji, self-rooted M9, and grafted Fuji/M9: (**A**) Fuji, (**B**) M9, (**C**) Fuji/M9, (**D**) flowering rate in 2017 and 2018, (**E**) shoot type in 2017, (**F**) shoot type in 2018.

Grafting plays a positive role in regulating the reproductive growth of scions [23,24]. The flowering rates in 2017 and 2018 were highest in M9, intermediate in Fuji/M9, and lowest in Fuji (Figure 1A–D). The flower buds of Fuji/M9 were in good condition in 2018 (Figure 1B), but less than 2% of the flower buds began to differentiate (Figure 1A,D). The flowering rate of M9 was 30% in 2017 and 40% in 2018 (Figure 1C,D). The number and percentage of long shoots were significantly lower in Fuji and Fuji/M9 than in M9 (Figure 1E,F). There were significantly fewer short shoots in Fuji than in M9 and Fuji/M9. There were more long branches in Fuji than in Fuji/M9, and the M9 and Fuji trees were tall and vigorous. Compared with Fuji, Fuji/M9 formed fewer shoots, had a lower proportion of long branches, and had a significantly higher proportion of short branches (Figure 1E,F).

The fresh weight of leaves in Fuji and Fuji/M9 increased from 30 DAFB (May 11) to 70 DAFB (July 11), and dropped off between 70 DAFB (July 11) and 153 DAFB (September 23), while in M9 it continuously increased from 30 DAFB (May 11) to 153 DAFB (September 23) (Table 1). The leaf area did not differ significantly among Fuji, Fuji/M9, and M9 in May, but was bigger in M9 than in Fuji and Fuji/M9 from 70 DAFB (July 11) to 153 DAFB (September 23) (Table 1).

### 2.2. Differences in Leaf and Bud Mineral and Sugar Contents among Different Apple Trees

**Figure 2:**
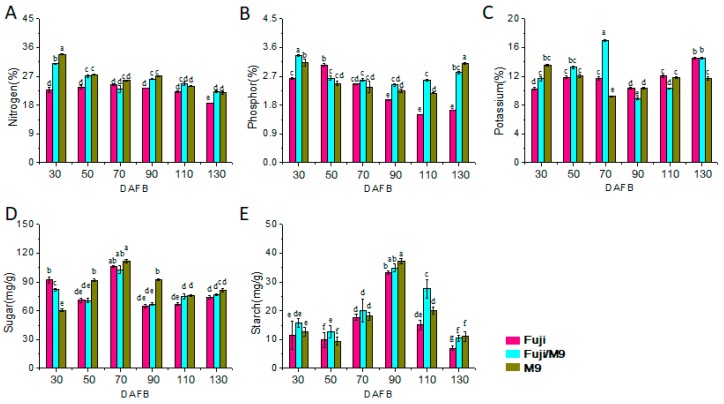
Total nitrogen (N), phosphorus (P), potassium (K), soluble sugar, and starch content of leaves in different apple trees: (**A**) total N content, (**B**) total P content, (**C**) total K content, (**D**) total soluble sugar content, and (**E**) starch content.

**Figure 3:**
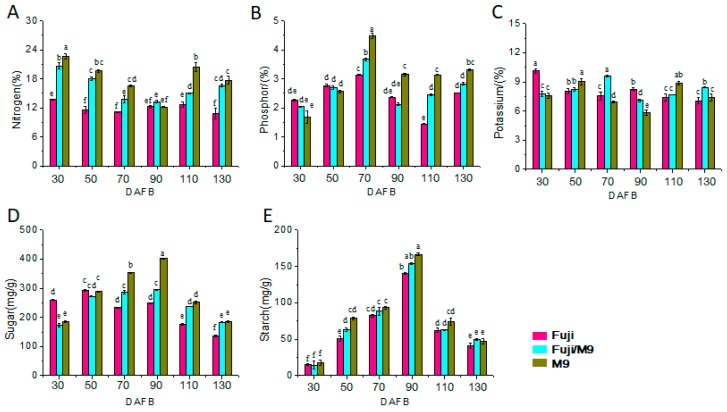
Total nitrogen (N), phosphorus (P), potassium (K), soluble sugar, and starch content of terminal buds in different apple trees: (**A**) total N content, (**B**) total P content, (**C**) total K content, (**D**) total soluble sugar content, and (**E**) starch content. Values followed by different lowercase letters mean significant at *p* < 0.05 levels.

To understand the role of grafting in the growth of Fuji on M9 rootstock, we determined the contents of total N, P, K, sugar, and starch in the leaves and buds (Figure 2 and Figure 3). The trends in the changes in N content in young leaves differed between Fuji and M9. In Fuji, the N content increased from 30 to 70 DAFB and then decreased from 70 to 130 DAFB. In M9, the N content decreased gradually from 30 to 130 DAFB. The total N content in young leaves of Fuji/M9 decreased from 30 to 70 DAFB, then increased from 70 to 90 DAFB, and then decreased from 90 to 130 DAFB (Figure 2A). The trends in the changes in N content in the buds were similar to those in leaves, but the N content was lower in buds than in leaves. The N content in the short shoots of Fuji and M9 first decreased, then increased, and finally decreased. The N content in buds increased from 70 to 110 DAFB in Fuji but increased from 90 to 110 DAFB in M9. In Fuji/M9, the N content in buds decreased from 30 to 90 DAFB, and then increased from 90 to 130 DAFB. The N content was always higher in Fuji/M9 than in Fuji (Figure 3A).

The P content in young leaves of Fuji increased from 30 to 50 DAFB, decreased continuously from 50 to 110 DAFB, and then increased slightly after 110 DAFB. The P content in M9 leaves decreased gradually from 30 to 110 DAFB, and then increased rapidly to a level higher than that on 0 DAFB. The trends in the change in P content in young leaves were similar in Fuji/M9 and M9; the P content first decreased and then increased. Except at 130 DAFB, the P content was always higher in Fuji/M9 than in M9 (Figure 2B). However, the P content in buds increased from 30 to peak at 70 DAFB in Fuji, Fuji/M9, and M9. The P content in buds of Fuji decreased from 110 to 130 DAFB, while that in M9 decreased rapidly from 70 to 90 DAFB and increased slightly from 90 to 130 DAFB. The trend in the change in P content was the same in Fuji/M9 and M9. After 70 DAFB, the P content in Fuji/M9 was lower than that in M9 and higher than that in Fuji (Figure 3B).

The trends in the change in K content in leaves differed between Fuji and M9. In Fuji, the K content increased from 30 to 50 DAFB, decreased from 50 to 90 DAFB, and then increased from 90 to 130 DAFB. In M9 (T337), the K content decreased from 30 to 70 DAFB, increased from 70 to 110 DAFB, and then slightly increased from 110 to 130 DAFB. In Fuji/M9, the K content gradually increased from 30 to 70 DAFB, decreased rapidly from 70 to 90 DAFB, and then increased from 90 to 130 DAFB (Figure 2C). The K content in buds of Fuji first decreased from 30 to 70 DAFB, then increased slightly from 70 to 90 DAFB, and decreased again from 90 to 130 DAFB. In M9, the K content in buds increased from 30 to 50 DAFB, decreased rapidly from 50 to 90 DAFB, increased rapidly from 90 to 110 DAFB, and then decreased from 110 to 130 DAFB. The K content in Fuji/M9 buds showed an N-shaped trend, increasing from 30 to 70 DAFB, decreasing from 70 to 90 DAFB, and then increasing from 90 to 130 DAFB. Except for 30 and 90 DAFB, the K content was higher in Fuji/M9 than in Fuji (Figure 3C).

The soluble sugar content in leaves of Fuji, Fuji/M9, and M9 peaked at 70 DAFB. The trend in soluble sugar content in the leaves of Fuji showed a W shape: a decrease from 30 to 50 DAFB, a rapid increase from 50 to peak at 70 DAFB, a rapid decrease from 70 to 90 DAFB, and then a slow increase from 90 to 130 DAFB. The soluble sugar content in M9 leaves showed an N-shaped trend: an increase from 30 to 70 DAFB, a decrease from 70 to 110 DAFB, and then a slow increase from 110 to 130 DAFB. The trend in soluble sugars in Fuji/M9 leaves was similar to that in Fuji leaves (Figure 2D). The trend in soluble sugar content differed between short apical buds and leaves. The soluble sugar content in short apical buds of Fuji slightly increased from 30 to 50 DAFB, quickly decreased from 50 to 70 DAFB, slightly increased from 70 to 90 DAFB, and then decreased from 90 to 130 DAFB. The soluble sugar contents in buds of M9 gradually increased from 30 to peak at 90 DAFB, then decreased from 90 to 130 DAFB. The soluble sugar content in buds of Fuji/M9 rapidly increased from 30 to 50 DAFB, then slowly increased from 50 to peak at 90 DAFB, and finally decreased slowly from 90 to 130 DAFB (Figure 3D).

The starch content showed the same trend in Fuji, Fuji/M9, and M9. It decreased slightly from 30 to 50 DAFB, increased from 50 to peak at 90 DAFB, and then decreased rapidly from 90 to 130 DAFB. At each time point, the starch content was higher in M9 than in Fuji. The starch content was higher in Fuji/M9 than in Fuji and M9 at 30, 50, 70, and 110 DAFB, and between that of Fuji and M9 at 90 and 130 DAFB (Figure 2E). In short apical buds of Fuji, starch increased from 30 to peak at 90 DAFB, and then decreased from 90 to 130 DAFB. At each time point, the starch content in buds was higher in M9 than in Fuji. The starch content in Fuji/M9 buds was between that of Fuji and M9 buds (Figure 3E).

### 2.3. Evaluation of miRNA Sequencing Data

**Figure 4:**
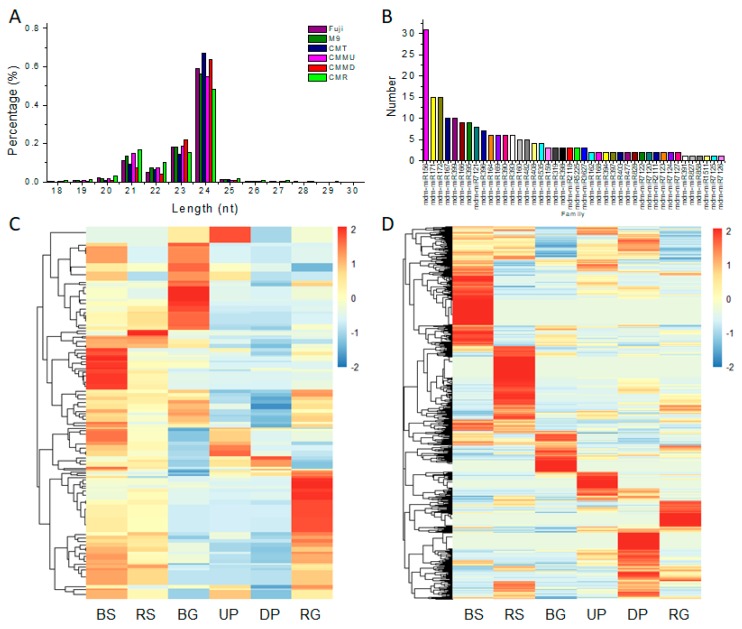
Overview of miRNAs in different apple trees. (**A**) Length distribution of identified miRNAs; (**B**) numbers of identified miRNAs in known miRNA families; (**C**) hierarchical clustering of identified known miRNAs; (**D**) hierarchical clustering of identified novel miRNAs. Branch tips of self-rooted Fuji (BS), root tips of self-rooted M9 (RS), branch tips of grafted Fuji/M9 (BG), root tips of grafted Fuji/M9 (RG), phloem of 10 cm above the graft union in Fuji/M9 (UP), phloem of 10 cm below the graft union in Fuji/M9 (DP).

To identify miRNAs playing an important role in grafting, six sRNA libraries were constructed from the following six tissues: (1) branch tips of Fuji (BS); (2) root tips of M9 (RS); (3) branch tips (BG), (4) root tips (RG), and (5) phloem of 10 cm above the graft union (UP); and (6) phloem of 10 cm below the graft union (DP) of Fuji/M9. The libraries were sequenced by the HiSeq2500 platform and greater than 11.6 million (M) clean reads were obtained for each sample (Table 2). Most sRNAs were 19–26 nt in length; the most abundant length was 24 nt, followed by 21 nt (Figure 4A). Our results are consistent with those of previously published reports [12].

To identify known miRNAs in apple, the sRNAs in the six bud libraries were used to query the miRbase 19.0 database (http://www.mirbase.org/) with the BLASTN program [25]. In total, we identified 206 known miRNAs belonging to 42 miRNA families (Figure 4B, Table S2). Some miRNA families had multiple members and some had only one member. The five miRNA families with the most members were miR156 (31 members), miR171 (15 members), miR172 (15 members), miR167 (10 members), and miR399 (10 members). Six miRNA families had only one member (miR391, miR827, miR858, miR1511, miR7125, and miR7126) (Figure 4B, Table S2).

### 2.4. Identification of Known and Novel miRNAs and Their Expression Profiles

In the six libraries, the expression levels of known miRNAs and novel miRNAs were calculated by their read count frequencies (Tables S2, S3). A row-scaled heatmap was used to cluster the known miRNAs according to their expression levels (Figure 4C). The known miRNAs clustered in 10 clades and some miRNAs were sample-specific. The 976 novel miRNAs were also subjected to hierarchical clustering and showed sample-specific clustering (Figure 4D, Table S3). The stem-loop hairpin structures of novel miRNAs precursors are summarized in Table S4.

**Figure 5:**
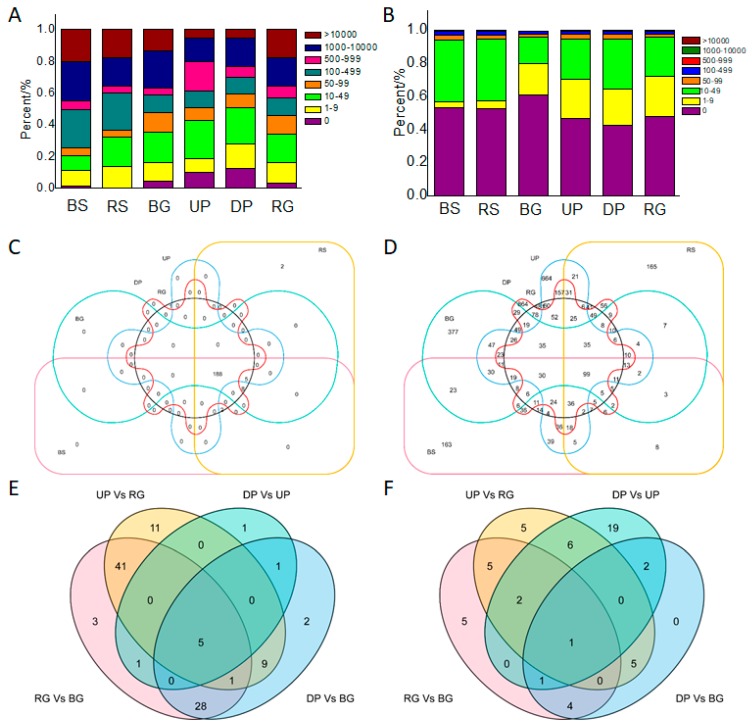
General information of miRNAs in different apple trees. (**A**) Expression levels of identified known miRNAs with their read content frequencies in each library; (**B**) expression levels of identified novel miRNAs with their read content frequencies in each library; (**C**) Venn analysis of identified known miRNAs; (**D**) Venn analysis of identified novel miRNAs; (**E**) Venn analysis of differentially expressed known miRNAs between BG, UP, DP, and RG; (**F**) Venn analysis of differentially expressed novel miRNAs between BG, UP, DP, and RG.

The known miRNAs in each sample were classified into eight categories based on their expression levels (read counts): level 0 (0 reads), level 1(1–9 reads), level 2 (10–49 reads), level 3 (50–99 reads), level 4 (100–499 reads), level 5 (500–999 reads), level 6 (1000–9999 reads), and level 7 (>10,000 reads) (Figure 5A) [2]. The ratios of known miRNAs in the eight categories varied among the six libraries (Figure 5A). The ratios were similar in the four libraries from Fuji/M9, and similar between the M9 and Fuji libraries. The largest percentages of known miRNAs in the BG, UP, DP, and RG libraries were in the level 3 (50–99 reads) category (19.42%, 23.79%, 22.82% and 18.45%, respectively). The largest percentages of known miRNAs in Fuji and M9 were in the level 4 (100–499 reads) category (23.79% and 23.79%, respectively). The lowest percentages of known miRNAs in the BG, UP, DP, and RG libraries were in the 0 (0 reads) category (4.85%, 10.19%, 12.62%, and 3.40%, respectively) (Figure 5A).

Novel miRNAs were also classified based on their expression levels (Figure 5B). The percentages of novel miRNAs were similar among the six libraries. The highest percentages of novel miRNAs in the BS, RS, BG, UP, DP, and RG libraries were in the 0 (0 reads) category (53.07%, 52.25%, 60.86%, 46.82%, 42.83% and 47.75%, respectively). The lowest percentage of novel miRNAs in the six libraries was in the level 7 (>10,000 reads) category (0.00% in all six libraries).

We constructed a Venn diagram to illustrate the expression patterns of known miRNAs in the six libraries (Figure 5C). In total, 188 out of 206 known miRNAs were shared among the six libraries; mdm-miR7123a and mdm-miR7123b were specifically expressed in M9 (Table S2). A Venn diagram was also constructed to illustrate the expression patterns of novel miRNAs (Figure 5D). Out of 976 novel miRNAs, 129 were shared among the six libraries. Many novel miRNAs were sample-specific. The numbers of novel miRNAs specific to each sample were as follows: 63 in BS, 53 in RS, 26 in BG, 20 in UP, 0 in DP, and 28 in RG.

### 2.5. Targets of Known miRNAs and Their Expression Patterns in Different Materials

To explore the differential expression of known miRNAs between different tissues of Fuji/M9, we compared miRNA expression between pairs of tissues (BG vs. DP, BG vs. RG, UP vs. DP, UP vs. RG) in Venn analyses (Figure 5E, Table 3). These analyses allowed us to identify the differentially expressed (up- or downregulated) known miRNAs between rootstocks and scions. Five members of the miR7121 family (mdm-miR7121d, mdm-miR7121e, mdm-miR7121f, mdm-miR7121g, and mdm-miR7121h) were differentially expressed between the rootstocks and scions. The target mRNAs of these five miR7121s were MD01G1113400, MD03G1107900, MD04G1105200, MD07G1180900, MD11G1121200, and MD12G1125900 (Table 3, Table S5). These target genes belonged to different gene families, suggesting that miR7121 is involved in various biological processes. Two other differentially expressed miRNAs were miR156 and miR172.

The novel miRNAs were analyzed in the same way to identify those differentially expressed between rootstocks and scions (Figure 5F). Only one novel miRNA, novel_mir_1174, was identified as being differentially expressed in all comparisons. There were 61 target genes of novel_mir_1174, and they were involved in multiple biological processes (Table S6).

**Figure 6:**
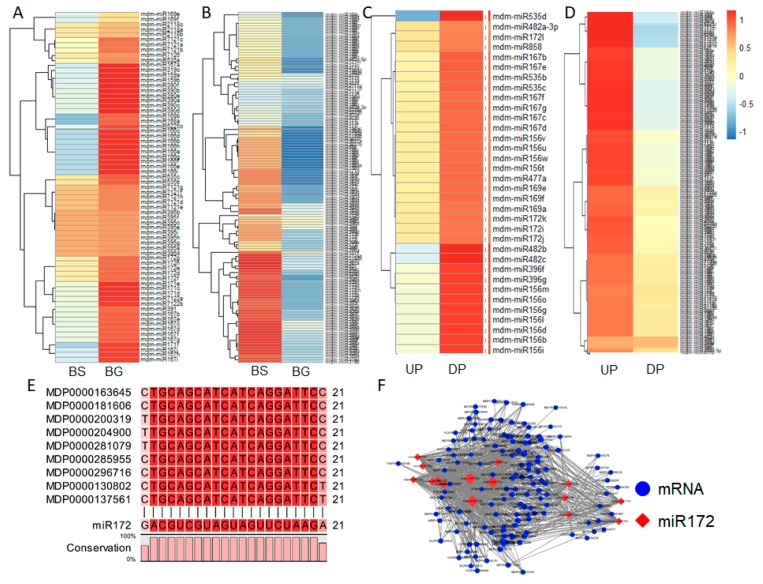
Heatmap analysis of differentially expressed miRNAs: (**A**) upregulated differentially expressed miRNAs in BS and BG; (**B**) downregulated differentially miRNAs in BS and BG; (**C**) upregulated differentially expressed miRNAs in UP and DP; and (**D**) downregulated differentially expressed miRNAs in UP and DP; (**E**) multiple alignment of *miR172* target sites of *AP2* gene family; (**F**) target network of miRNA172 family with its target genes.

To generate a comprehensive view of grafting-responsive miRNAs, we filtered differentially expressed miRNAs between BS and BG. Figure 6A,B show the upregulated and downregulated known miRNAs, respectively, in BG compared with BS (Table S7). The upregulated miRNAs included five miR172 family members (Figure 6A), and the downregulated miRNAs included 31 miR156 family members (Figure 6B). We also identified the differentially expressed miRNAs between UP and DP, and identified 11 miR172 family members that were upregulated in UP compared with DP (Figure 6C), and 11 miR156 family members that were downregulated in UP compared with DP (Figure 6D, Table S7). It has been suggested that miR172 plays a positive role in regulating flowering. The target genes of miR172 were extracted and aligned. The target genes of miR172 were members of the AP2 gene family and the target sites were highly conserved (Figure 6E). An miRNA172s–mRNA network was constructed using Cytoscape. The network was complex, suggesting that the miRNA172s were functionally diverse (Figure 6F).

**Figure 7:**
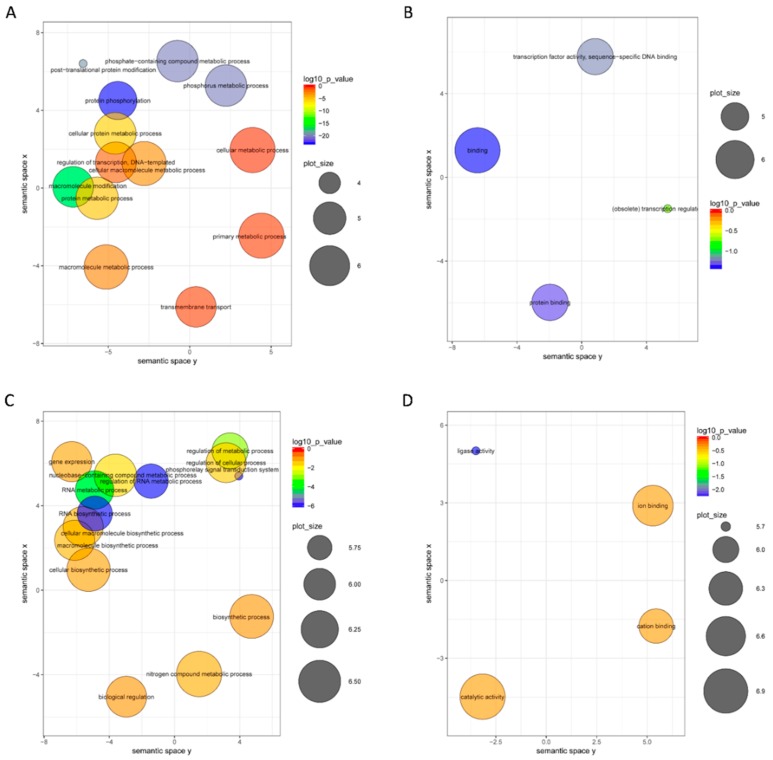
GO enrichment analysis targeting genes of differentially expressed miRNAs in BG and BS. (**A**) Biological process of GO enrichment analysis for upregulated miRNA target genes; (**B**) molecular function of GO enrichment analysis for upregulated miRNA target genes; (**C**) biological process of GO enrichment analysis for downregulated miRNA target genes; (**D**) molecular function of GO enrichment analysis for downregulated miRNA target genes.

To identify the biological processes that differed between BS and BG, target genes of differentially expressed miRNAs were subjected to a GO enrichment analysis with a focus on the biological process and molecular function categories (Figure 7). In the biological process category, the targets of upregulated miRNAs were mainly related to the phosphorus metabolic process and posttranslational protein modification (Figure 7A). In the molecular function category, the targets of upregulated mRNAs were mainly related to DNA and protein binding (Figure 7B). In the biological process category, the targets of downregulated miRNAs were mainly involved in the RNA metabolic process (Figure 7C). In the molecular function category, the targets of downregulated miRNAs were mainly related to iron binding and catalytic activity (Figure 7D).

### 2.6. qRT-PCR Analysis of Candidate Differentially Expressed Genes

**Figure 8:**
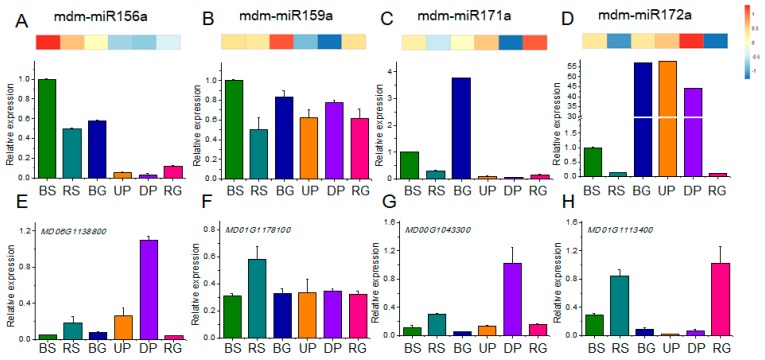
qRT-PCR analysis of differentially expressed miRNAs and their target genes in different samples. (**A**–**D**) miRNA expression profiles; upper heatmap shown with sequencing data, bottom histogram shown with qRT-PCR; (**E**–**H**) qRT-PCR analysis of expression profiles of their target genes in different samples.

To confirm the miRNA sequencing data, we quantified four differentially expressed miRNAs (miRNA156, miRNA159, miRNA171, and miRNA172) in different tissues (BS, RS, BG, UP, DP, and RG) of Fuji, M9, and Fuji/M9 (Figure 8A–D). The expression levels of these four miRNAs differed among the six tissues, consistent with the sequencing data. For example, miR156 was present at higher levels in BS than in other tissues in both the sequencing and qRT-PCR results (Figure 8A), while with analysis expression of miR159 and miR172 (Figure 8B,D), little differences were noticed between sequencing and qRT-PCR results. For example, miR172 was highest in GDP in the sequencing data, and highest in BG from qRT-PCR results (Figure 8D). We also analyzed the transcript levels of the miRNA target genes by qRT-PCR (Figure 8E–H). All the predicted targets showed expression trends opposite to those of their targeting miRNAs. For example, in DP, there were low levels of miRNA156 and high transcript levels of its target, *SPL* (*MD06G1138800*) (Figure 8E). Similarly, in RS, miR159 showed low expression levels while its target, *MYB* (MD01G1178100), showed high transcript levels (Figure 8F). The same trend was observed for miR171 and *GRAS* (MD00G104300) in BG, and for miR172 and *AP2* (MD01G1113400) in RG (Figure 8G,H).

**Figure 9:**
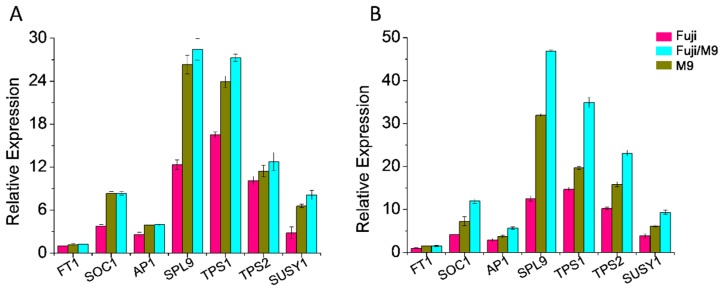
qRT-PCR analysis of relative expression levels of flowering related genes in leaves and flower buds in Fuji, Fuji/M9, and M9: (**A**) leaves, (**B**) buds.

Because Fuji, Fuji/M9, and M9 had different flowering phenotypes, we also quantified the transcript levels of several flowering-related genes in tissues from 30 DAFB. In the leaves, all genes showed the highest transcript levels in M9. The transcript levels of suppressor of overexpression of CONSTANS 1 (*MdSOC1*), APETALA1 (*MdAP1*), and sucrose synthase 1 (*MdSUSY1*) were similar in M9 and Fuji/M9, and were significantly higher in M9 and Fuji/M9 than in Fuji. The transcript levels of flowering locus T (*MdFT1*) and trehalose-6-phosphate synthase 2 (*MdTPS2*) were significantly higher in M9 than in Fuji. The transcript levels of SQUAMOSA promoter-binding protein-like 9 (*MdSPL9*), *MdTPS1*, and *MdTPS2* were highest in M9 and lowest in Fuji (Figure 9A). In flower buds, the transcript levels of all analyzed genes except for *MdFT1* were higher in M9 than in Fuji and Fuji/M9. The transcript levels of *MdSOC1*, *MdSPL9*, *MdTPS1*, *MdTPS2*, *MdSUSY1*, and *MdAP1* were significantly higher in Fuji/M9 than in Fuji (Figure 9B).

## 3. Discussion

Use of dwarf trees and close planting are general trends in the cultivation of fruit trees around the world [26]. M9 is a popular dwarf rootstock that is widely used in apple cultivation. The rootstock, the scion, and their interaction affect fruit quality, plant resistance, and yield, which are important factors for economic efficiency [27]. In this study, we analyzed three different materials, self-rooted Fuji, self-rooted M9, and grafted Fuji/M9, to investigate their morphologic, physiologic, and molecular differences. Although various different rootstocks and scions are used in the apple industry, our basic investigation of M9 together with Fuji could also be useful for further analysis. These results provide valuable information and identify factors involved in the interaction between scions and rootstocks, which could be useful in apple as well as other fruit trees.

### 3.1. Flowering Rate and N, P, K, and Soluble Sugar Contents Are Affected by Grafting

The key factor in achieving high yield and stable production of fruit trees is the ability to form a sufficient number of flower buds. An ample supply of nutrients can enhance the formation of flower buds in fruit trees [28]. Flower induction is a complex morphogenic process that requires large amounts of nutrients and minerals. In particular, N, P, and K are indispensable for flower induction. Previous studies have shown that endogenous hormones, nucleic acids, carbohydrates, and proteins also affect flower induction. Leaves are the main organs for producing nutrients in the plant and are the main source of nutrients for flower induction [29,30,31]. In the present study, we found significant differences in fresh weight, dry weight, and leaf area among the three materials, indicating that these attributes were affected by grafting.

In the present study, the Fuji trees showed strong growth but formed few flowers. However, Fuji grafted onto M9 rootstock formed more short branches, which was indicative of a better balance between vegetative growth and reproductive growth, and showed enhanced flower induction (Figure 1C). The contents of mineral elements such as N, P, and K are related to plant species and age, and differ among various tissues and organs. Flower formation and quality are affected by N, P, and K contents [32]. The contents of N-containing substances in leaves and buds can significantly affect the differentiation of flower buds [33]. The N content in leaves and buds was higher in Fuji/M9 than in Fuji (Figure 2A and Figure 3A). Interestingly, the N content gradually decreased in M9, while it gradually increased in Fuji/M9, indicating that N might be a critical nutrient mobilized from the rootstock to the scion. The process of plant flower bud differentiation is also affected by P and K [34]. In the present study, the contents of P and K were significantly higher in Fuji/M9 than in Fuji (Figure 2B,C and Figure 3B,C).

The flower bud formation process in fruit trees includes two periods: physiologic differentiation and morphologic differentiation. The changes during physiologic differentiation are not visible. The main feature of this process is that a series of flowering genes are activated, and flower buds no longer revert to nutritional buds [35]. Grafting has been shown to affect the transport of nutrients and water [28]. In our study, there were higher contents of N, P, and K, and soluble sugars in the buds and their adjacent leaves in grafted Fuji/M9 dwarfing rootstocks than in M9 or Fuji, which resulted in an increased number of flower buds and a higher flowering rate in Fuji/M9.

### 3.2. Grafting-Induced miRNAs Involved in Bud Growth and Flower Induction

Flower induction is a very complicated process that is regulated by at least six pathways in *Arabidopsis*: the photoperiod, vernalization, temperature sensitivity, autonomy, gibberellin, and age pathways. These pathways constitute a complex genetic network. From flower induction to the formation of floral organs, miRNAs play a key role in the floral development process [36]. Various miRNAs, such as miR156 and miR172, and transcription factors, such as *AP2* and *SOC1*, are involved in floral induction and development [36,37]. miR172 and miR156/157 are involved in regulating the transition from vegetative to reproductive growth [37]. In *Arabidopsis*, miR156*–SPL* has an important regulatory role in the transition from vegetative to reproductive growth [18]. miR172 and miR169 regulate floral organ properties by defining the regions of target gene expression during the early stages of floral development. We detected higher expression of miR169 in the branch tips of Fuji/M9 than Fuji (Figure 6A), consistent with the important role of miR169 in regulating flower induction [38]. In shoot tips, the grafted plants had higher levels of miR2118 and miR7121, indicative of their role in the grafting response [39,40].

In *Arabidopsis*, members of the miR156 family posttranscriptionally regulate the expression of the SPL gene family [16,41], whose members are involved in floral induction. miR319, miR159, miR164, and miR167 are also involved in floral development. The late stage of floral development determines cell specialization [41]. miR156 targets the SPL3 gene family, whose members activate the expression of the flowering pathway integrator genes *LFY*, *FUL,* and *AP1* to induce flowering. SPL9 and SPL15 induce the expression of miR172, which indirectly promotes flowering [37,41]. In *Arabidopsis*, miR172 regulates six *AP2* flowering repressor genes (*AP2*, *TOE1*, *TOE2*, *TOE3*, *SMZ,* and *SNZ*), all of which contain miR172 binding sites. The AP2 gene family shows high functional redundancy, as overexpression of any one of the *AP2* genes caused a late-flowering phenotype. Plant lines overexpressing miR172 or mutants with loss of *TOE1* showed early flowering phenotypes [42]. Overexpression of miR172 resulted in a decrease in the abundance of AP2. miR156 and miR172 interact to form a feedback loop that regulates reproductive growth in plants [43]. In the present study, miR172 showed higher expression levels in the branch tips of Fuji/M9 than in Fuji. Also, miR172 was expressed at higher levels in the phloem above the graft union than in the phloem below the graft union. Previous studies have reported that some miRNAs, including miR339, miR172, and miR395, are transported between the scion and rootstock [13,44,45]. Considering the differences in expression profiles near the graft union, we concluded that miR172 could be transported between the M9 rootstock and the Fuji scion. However, because the sequence of miR172 is the same in M9 and Fuji, we could not distinguish which part miR172 originated from. Furthermore, grafting may also cause changes in expression. The origin and mechanism of miR172 still need to be verified.

**Figure 10:**
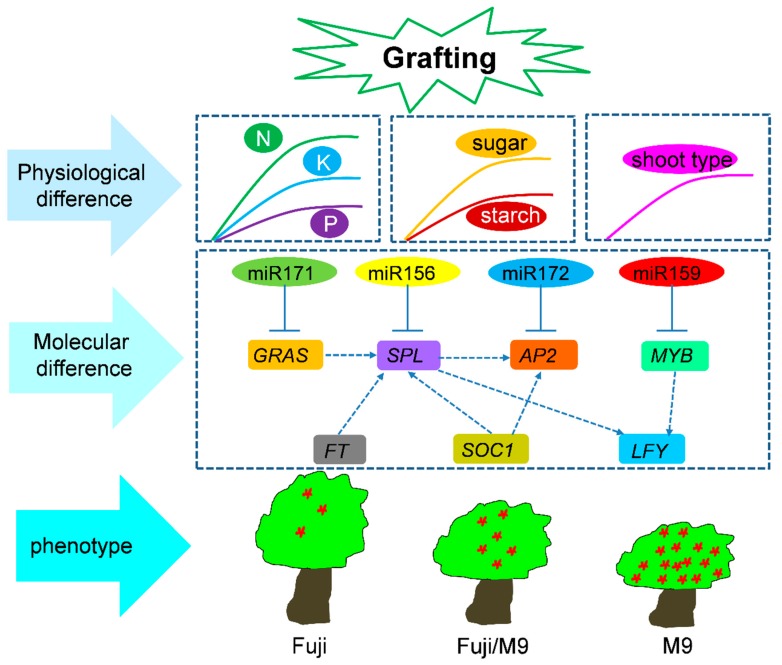
Hypothetical model for critical regulatory networks in response to grafting processes between scions and rootstocks in *Malus*.

We identified five *MYB* genes as targets of miR159 and six *GARS* genes as targets of miR171 (Table 3). The trends in the expression of these miRNAs and their targets were verified by qRT-PCR, and the results confirmed the opposite expression patterns of these miRNAs and their targets (Figure 8). The target genes of miR156, miR159, miR171, and miR172 were transcription factors involved in regulating flower formation. The interaction between miRNAs and mRNAs indicated that a complex regulatory network was induced by grafting (Figure 10).

### 3.3. Regulatory Network of miRNAs–mRNAs in Induction of Flowering

The apical meristems of higher plants are transformed into inflorescence meristems under appropriate external conditions. In flowering meristems, the differentiation of flower organ primordia begins after the floral meristem has differentiated, resulting in the formation of various floral organs [3].

*SPL* transcription factors can directly or indirectly regulate the flowering time of plants by participating in photoperiodic, gibberellin, and age-related pathways. The *SPL* genes regulate plant flowering by directly activating downstream floral meristem-specific genes, such as *LEAFY (LFY)* [19]. In the present study, MD06G1138800 (*SPL*) was upregulated in the branch tips of Fuji/M9 compared to Fuji (Figure 8A), indicating that grafting could increase the expression of *SPL* and promote flower formation. Genes in the AP2 family show high functional redundancy, and constitutive expression of any one of the *AP2* genes has been shown to cause a late-flowering phenotype [43]. Flower formation is negatively regulated by AP2, which is targeted by miR172. We found that miR172 was expressed at high levels in in the branch tips of Fuji/M9, resulting in the inhibition of *AP2* expression (Figure 8D).

*MdSOC1* and *MdFT* are core genes in the flowering-inducible pathway [46]. In short shoot buds and leaves, the expression levels of *MdSOC1* and *MdFT* were higher in Fuji/M9 than in Fuji, consistent with the higher flower formation rate in Fuji/M9 (Figure 8). The product of *MdSOC1* can integrate diverse signals such as photoperiod, temperature, hormones, and life cycle, which are coordinated by two antagonistic flowering genes, *MdCO* and *MdFLC* [47]. The floral meristem gene *MdAP1* is known to be regulated by *MdSOC1*. The expression level of *MdAP1* was similar to that of *MdSOC1* in leaves and short shoots.

In the present study, our results revealed different levels of varieties after grafting. First, at the physiologic level, N, P, K, sugar, and starch content, as well as shoot type, were significantly different in grafted apple trees. Then, we verified the regulatory network between miRNA and mRNA related to flower induction. Even through the regulatory network has been discussed in other species, our experiments indicated dynamic interactions of the miRNAs, and mRNAs were affected by grafting. Finally, the phenotype of Fuji was greatly changed after being grafted onto M9 (Figure 10). However, the connections between the three levels still need further study.

In summary, after grafting onto M9 dwarf rootstock, Fuji showed improvements in leaf quality and higher mineral contents, carbohydrate contents, and transcript levels of flowering-related genes. From both a physiologic and molecular perspective, grafting can increase the number of flowers in Fuji when grafted onto M9.

## 4. Materials and Methods

### 4.1. Plant Materials

These experiments were conducted in the Apple Demonstration Nursery of Yangling Modern Agriculture Technology Park (Northwest Agriculture and Forestry University), Shaanxi Province, China (108°04′ E, 34°16′ N). Three apple materials were analyzed: M9, Fuji, and Fuji/M9. Twenty-seven 3-year-old trees were selected for each combination, making a total of 81 trees. The samples included branch tip from self-rooted scions (BS) (Figure 1A), root tip from self-rooted rootstock (RS) (Figure 1B), branch tip from grated combinations (BG), root tip from grated combinations (RG), up 10 cm part of the stem above the graft union (UP), and down 10 cm part of the stem above the graft union (DP) (Figure 1C). These samples were collected on 13 May 2017 (30 days after full blossom, DAFB), when flower induction began, and stored in liquid nitrogen until analysis. Flowering rate and shoot types were calculated according to a previous study [12].

### 4.2. Determination of Total Nitrogen, Phosphorus, and Potassium Contents

Apical buds and adjacent leaves on the apical buds with no pests were collected on day (d) 30, 50, 70, 90, 110, and 130 after flowering in 2017. Plant materials were brought back to the laboratory in an ice box, cleaned, and wiped dry. The samples were oven-dried at 105 °C for 20 min, and then at 80 °C until they reached constant weight. The dried samples were ground to pass through a 60 mesh (diameter 0.25 mm) nylon sieve. Three replicates per sample were prepared and analyzed. The total nitrogen (N), phosphorus (P), and potassium (K) contents were determined as described previously [48].

### 4.3. Determination of Soluble Sugar and Starch Contents

Plant materials from the above dates were dried in a baking oven and ground into a powder. A part of each sample (0.3 g) was added to a 10 mL tube. For the soluble sugar determination, 7 mL of 80% ethanol was added to the tube and the mixture was shaken at 70 °C in a water bath for 30 min. The sample was centrifuged at 8000 rpm for 10 min and the supernatant was transferred to a new tube containing 11 mL of 80% ethanol. The mixture was shaken vigorously and then centrifuged. The supernatant was completed to a volume of 20 mL. Finally, the solution was dried, dissolved in ddH_2_O, and then mixed with anthrone reagent, according to a previous study [49]. The optical density (OD) was measured at 620 nm [50].

To quantify starch, dried plant powder was gelatinized in a boiling water bath for 10 min. After cooling to room temperature, 2 mL of 9.2 mol/L perchloric acid was added, the mixture was stirred for 15 min, and then 10 mL of ddH_2_O was added. The mixture was centrifuged at 8000 rpm for 10 min. The supernatant was collected and completed to a volume of 50 mL. The starch content was determined by titration [50].

### 4.4. Construction and Sequencing of sRNA Libraries

Total RNA was isolated from leaves, buds, and root tips using an E.Z.N.A.^®^ Plant RNA Kit (Omega Bio-Tek, Doraville, GA, USA), and then sequenced on a HiSeq sequencer (Illumina, San Diego, CA, USA) at the Beijing Genomics Institute (BGI), Shenzhen, China. The procedure was as follows: after the small RNAs (sRNAs) (18–30 nt long) were separated and purified on a 15% tetra bromoethane-urea (TBE-urea) denaturing polyacrylamide gel, they were ligated with 5′ and 3′ adaptors. Then they were reversed-transcribed into single-stranded cDNAs and amplified to generate double-stranded cDNAs. The cDNAs were sequenced.

### 4.5. Analysis of High-Throughput Sequencing Data

The raw sequences were initially processed by BGI and filtered to obtain clean reads. Fragments between 18 and 30 nt in length were mapped to the apple genome (https://www.rosaceae.org/) using SOAP2 [51]. We annotated the rRNAs, scRNAs, snoRNAs, snRNAs, and tRNAs by BLASTN searches against the National Center for Biotechnology Information (NCBI) GenBank and Rfam databases. The remaining sequences were used in BLAST searches against miRbase 22.0 (http://www.mirbase.org/) to identify conserved and known miRNAs in apple. The target genes of known miRNAs in apple were predicted with MIREAP software (http://sourceforge.net/projects/mireap/). Novel miRNAs were identified as described elsewhere [5]. The stem-loop RNA structures of novel miRNAs were predicted using RNAfold.

Gene Ontology (GO) enrichment analyses were performed using AgriGO [52]. The generated raw GO enrichment files were modified using R software. The miRNA–mRNA network was constructed using Cytoscape version 3.5.1.

### 4.6. Verification of Expression of Differentially Expressed miRNAs and Their Predicted Targets by qRT-PCR

First-strand cDNA was synthesized using a Prime Script™ RT reagent kit (TaKaRa, Otsu, Japan). Primers were designed based on the gene sequences in the Golden Delicious Apple Genome Database (https://www.rosaceae.org/) (Supplementary Table S1) [53]. The relative expression level of detected genes was calculated with the 2^−ΔΔ*C*t^ method using the apple *MdActin* gene (GenBank number: GQ339778) as an internal reference [54,55].

First-strand cDNA was synthesized using a miRcute miRNA cDNA synthesis reagent kit (Tiangen, Beijing, China) for miRNA qualification. Primers were designed according to the instructions in the user manual. The relative expression levels of miRNAs were calculated as described elsewhere [56].

## Figures and Tables

**Table 1:** Morphologic differences leaves of different apple combinations. DAFB, days after full blossom.

Index	Cultivar	Sampling Time		
		May 11, 30 DAFB	July 11, 70 DAFB	Sept. 23, 153 DAFB
Fresh weight (FW) (g)	Fuji	52.467 ± 0.321 ^b^	59.633 ± 1.793 ^b^	56.400 ± 5.950 ^c^
	Fuji/M9	53.600 ± 4.178 ^b^	79.167 ± 1.074 ^a^	75.167 ± 0.500 ^b^
	M9	57.700 ± 6.146 ^b^	77.300 ± 1.639 ^a^	89.767 ± 0.560 ^a^
Dry weight (DW) (g)	Fuji	18.447 ± 0.185 ^bc^	21.000 ± 1.472 ^c^	20.576 ± 2.210 ^d^
	Fuji/M9	17.533 ± 1.330 ^c^	28.252 ± 1.045 ^b^	29.208 ± 0.430 ^c^
	M9	20.933 ± 2.332 ^ab^	29.433 ± 1.222 ^b^	37.300 ± 1.351 ^a^
DW/FW	Fuji	35.161 ± 0.392 ^a^	35.215 ± 1.612 ^c^	36.662 ± 0.845 ^d^
	Fuji/M9	32.717 ± 0.285 ^b^	35.681 ± 0.940 ^c^	38.857 ± 0.534 ^c^
	M9	36.267 ± 0.752 ^a^	38.083 ± 1.577 ^b^	41.550 ± 1.408 ^b^
Leaf area (cm^2^)	Fuji	3127.546 ± 36.797 ^a^	5022.645 ± 97.551 ^b^	6476.498 ± 115.639 ^b^
	Fuji/M9	3007.270 ± 58.385 ^a^	4879.042 ± 73.521 ^b^	6364.967 ± 139.101 ^b^
	M9	3100.655 ± 51.934 ^a^	5938.932 ± 108.548 ^a^	9090.272 ± 155.033 ^a^

Values in the same column followed by different lowercase letters mean significant at *p* < 0.05 levels.

**Table 2:** Statistical analysis of sequencing reads for six sRNA libraries. BS, branch tips of Fuji; RS, root tips of M9; BG, branch tips and RG, root tips of 10 cm above the graft union (UP); DP, phloem of 10 cm below the graft union.

Types	Read Counts					
	BS	RS	BG	UP	DP	RG
Clean reads	11,686,623	11,590,477	11,775,098	11,874,809	11,663,829	11,896,826
Reads mapped to genome	3,667,388	3,335,867	3,580,308	3,546,267	3,756,158	2,954,069
rRNA snRNA snoRNA tRNA	637,318	457,197	367,837	278,159	427,007	540,889
miRNA	434,003	256,294	139,371	236,376	194,101	326,400

**Table 3:** Potential targets of identified known miRNAs in apple (*Malus domestica* Borkh.) and their GO biological process.

Known miRNAs	Targets			
	Gene ID	GO	Arabidopsis ID	Description
miR156	MD06G1138800	GO:0003677, GO:0005634	AT5G50670.1	SPL
	MD06G1204000	GO:0003677, GO:0005634	AT1G69170.1	
	MD07G1111600	GO:0003677, GO:0005634	AT1G27360.1	
	MD09G1008900	GO:0003677, GO:0005634	AT5G50670.1	
	MD12G1060200	GO:0003677, GO:0005634	AT2G42200.1	
	MD13G1059500	GO:0003677, GO:0005634	AT1G69170.1	
	MD13G1120300	GO:0003677, GO:0005634	AT5G43270.1	
	MD14G1060200	GO:0003677, GO:0005634	AT2G42200.1	
	MD14G1215300	GO:0003677, GO:0005634	AT1G69170.1	
	MD16G1058800	GO:0003677, GO:0005634	AT1G69170.1	
	MD16G1122000	GO:0003677, GO:0005634	AT5G50670.1	
	MD17G1001400	GO:0003677, GO:0005634	AT5G50670.1	
miR159	MD01G1178100	GO:0003677	AT3G60460.1	MYB
	MD07G1085000	GO:0003677	AT3G11440.1	
	MD07G1101000	GO:0003677	AT3G60460.1	
	MD11G1215600	GO:0003677	AT4G26930.1	
	MD11G1216000	GO:0003677	AT2G32460.1	
miR171	MD00G1043300		AT4G00150.1	GRAS
	MD02G1210200		AT4G00150.1	
	MD04G1088100		AT4G00150.1	
	MD07G1115800		AT2G45160.1	
	MD08G1167600		AT4G08250.1	
	MD15G1353200		AT4G08250.1	
miR172	MD01G1113400	GO:0003700, GO:0006355	AT2G28550.1	AP2
	MD03G1107900	GO:0003700, GO:0006355	AT2G28550.3	
	MD04G1105200	GO:0003700, GO:0006355	AT2G28550.3	
	MD07G1180900	GO:0003700, GO:0006355	AT2G28550.2	
	MD11G1121200	GO:0003700, GO:0006355	AT2G28550.3	
	MD12G1125900	GO:0003700, GO:0006355	AT2G28550.3	

## Supplementary Materials

The following are available online at http://www.mdpi.com/1422-0067/19/8/2384/s1.
